# A study of the factors associated with emergency department visits in advanced cancer patients receiving palliative care

**DOI:** 10.1186/s12904-022-01098-w

**Published:** 2022-11-15

**Authors:** Netsakao Dumnui, Kittiphon Nagaviroj, Thunyarat Anothaisintawee

**Affiliations:** Department of Family Medicine, Faculty of Medicine, Ramathibodi Hospital, Mahidol University, Bangkok, Thailand

**Keywords:** Palliative care, Cancer, Emergency room, Emergency department, Visit, ED, ER

## Abstract

**Purpose:**

Several studies demonstrated that cancer patients visited the emergency department (ED) frequently. This indicates unmet needs and poor-quality palliative care. We aimed to investigate the factors that contribute to ED visits among patients with advanced cancer in order to identify strategies for reducing unnecessary ED visits among these patients.

**Methods:**

A retrospective study was conducted between January and December, 2019. Eligible patients were previously enrolled in the comprehensive palliative care program prior to their ED visit. All patients older than 18 were included. Patients were excluded if they had died at the initial consultation, were referred to other programs at the initial consultation, or had an incomplete record. The trial ended when the patients died, were referred to other palliative programs, or the study ended. The time between the initial palliative consultation and study endpoints was categorized into three groups: 16 days, 16–100 days, and > 100 days, based on the literature review. To investigate the factors associated with ED visits, a logistic regression analysis was conducted. The variables with a *P* value < 0.15 from the univariate logistic regression analysis were included in the multiple logistic regression analysis.

**Results:**

Among a total of 227 patients, 93 visited the ED and 134 did not. Mean age was 65.5 years. Most prevalent cancers were colorectal (18.5%), lung (16.3%), and hepatobiliary (11.9%). At the end, 146 patients died, 45 were alive, nine were referred to other programs, and 27 were lost to follow-up. In univariate logistic regression analysis, patients with > 100 days from palliative consultation (OR 0.23; 95%CI 0.08, 0.66; *p*-value 0.01) were less likely to attend the ED. In contrast, PPS 50–90% (OR 2.02; 95%CI 1.18, 3.47; *p*-value 0.01) increased the ED visits. In the multiple logistic regression analysis, these two factors remained associated with ED visits:> 100 days from the palliative consultation (OR 0.18; 95%CI 0.06, 0.55; *p*-value 0.01) and PPS 50–90% (OR 2.62; 95%CI 1.44, 4.79; *p*-value 0.01).

**Conclusions:**

There was reduced ED utilization among cancer patients with > 100 days of palliative care. Patients having a lower PPS were associated with a lower risk of ED visits.

## Background

The frequent emergency department (ED) visits of patients with advanced cancer indicated unmet palliative care needs and the low quality of palliative care at the end of life [1]. Generally, patients and families describe these visits as unpleasant experiences [2]. This can be attributed to several factors, including long wait times due to their patients’ ranking second to critically ill patients, a lack of adequate symptom palliation, ineffective communication due to the noisy and impersonal environment, and a lack of dedicated space for relatives to wait for examinations or a place to discuss advance care planning during the final stages of life [2, 3]. Significant barriers to providing appropriate palliative care in the ED include the absence of long-term relationships between ED physicians and patients; insufficient staff training in symptom management; and essential communication skills, such as discussing the goal of care and advance care planning [4–6].

Multiple previous studies have demonstrated that patients with advanced cancer have a high rate of emergency department visits. A Canadian study revealed that 76,759 patients visited the emergency department 194,017 times during their final 6 months of life [7]. Additionally, some studies discovered that between 30.7 and 60% of terminal cancer patients visited an emergency department during their last month of life [8–11]. According to studies, a variety of factors contribute to people with advanced cancer seeking care in the emergency department, including uncontrolled symptoms (such as uncontrolled pain, dyspnea, fatigue, and confusion) [12], incompetent caregivers, concerns or fear about the disease itself, a sense of security when receiving care in a hospital, and difficulty accessing health care facilities in the community, particularly when symptoms change abruptly or occur outside of normal office hours [13, 14]. According to numerous studies, the majority of patients visit emergency departments outside of business hours or when community palliative care is unavailable [14, 15]. Although visiting the emergency room is not always the best option for certain patients. However, as the disease progresses and the patient is unable to manage the symptoms at home, admission to the emergency department may become necessary.

It is worth noting that nearly one-fourth to half of ED visits by patients with advanced cancer receiving palliative care were potentially avoidable [16, 17]. Early palliative care, including advance care planning and anticipatory guidance on symptom management, may assist patients and caregivers in preparing for disease progression, reducing unnecessary emergency department visits and enhancing the quality of life of cancer patients, according to additional evidence [13, 18, 19]. A study found that terminally ill cancer patients who were referred to palliative care at least 3 months prior to death had fewer visits to the emergency department than those who were referred late [20].

As end-of-life ED visits for advanced cancer patients are avoidable. The review of the literature revealed a lack of research on the factors associated with ED visits among cancer patients receiving palliative care. Gaining a deeper understanding of the factors that can prevent ED visits in patients with advanced disease can improve palliative care. This study’s objective was to investigate the factors that contribute to ED visits among patients with advanced cancer in order to identify strategies for reducing unnecessary ED visits among these patients.

## Methods

We conducted a retrospective cohort study on cancer patients receiving palliative care by reviewing their medical records and electronic databases. All patients who were referred to our palliative care program between January 1, 2019 and December 31, 2019 and met the inclusion criteria were tracked and recorded the number of ED visits that occurred following the referral. The patients were evaluated and continued to be followed up by a palliative care team until the study’s endpoints, which were death, referral to other palliative care programs, or December 31, 2019.

### Inclusion and exclusion criteria

All patients with a cancer diagnosis who registered for our palliative care program and were over the age of 18 were included. Patients were excluded if they died prior to hospital discharge during the initial ED or inpatient consultation, if they were referred to other palliative care programs at the initial consultation, or if their medical records had incomplete data.

### Palliative care programme

Since 2010, the Faculty of Medicine at Ramathibodi hospital, Mahidol University, has been developing palliative care services for Bangkok metropolitan area residents. The project aims to integrate palliative care services into conventional medicine by providing inner-city residents a variety of services, such as inpatient consultation, a palliative care clinic, a palliative care unit, telephone consultation, and home-based palliative care. Approximately 80% of the roughly 500 new patients and their families who participated in this project each year had a cancer diagnosis.

Primary physicians, such as oncologists, surgeons, radiation oncologists, and gynecologists, identified the cancer patients who required palliative care and made the referral. The program may be entered by inpatient consultation, outpatient consultation, or emergency department consultation. After the palliative consultation, a coordinator of palliative care inputted the patient’s information into a database program to register the patient. Patients were then followed by a palliative care team consisting of palliative care physicians, nurses, case managers, and health alliances across the continuum of palliative care services, from the initial consultation until the patient’s death or referral to other palliative care programs.

The palliative care team constructed a standard data record to store the clinical information pertaining to the provision of palliative care, including symptom scores assessed by the Thai version of the Edmonton Symptom Assessment System, Palliative Performance Scale, psychosocial information, advance care planning discussion, and advance directive. For each consultation, the palliative care physician on the team completed the form.

During non-office hours, the same palliative care team provided emergency department consultation on symptom management, advance care planning, and discharge planning. Each patient received a weekly routine follow-up contact from a case manager or team member, who updated clinical information in the patient’s medical record. The team would provide palliative home care based on the needs of individual patients. If the patient dies, the case manager would also coordinate bereavement care.

### Ethics

The Human Research Ethics Committee of the Faculty of Medicine, Ramathibodi Hospital, Mahidol University has reviewed and approved this study with the code COA. MURA2021/184. All methods were carried out in accordance with the approved study protocol under the standard regulations and the Declaration of Helsinki. The participants’ confidentiality is anonymized. The research data is backed up on two computers with passwords and then analyzed and summed up as a whole so that no one can be identified.

### Data collection

The data for the study was gathered using a standardized data collection form. The principal investigator extracted the data from medical records and electronic databases and then entered the data into the form. A second researcher cleaned and checked the accuracy of data entry using techniques such as visual inspection and the search for outliers or unnatural data deviations. Those two researchers had a meeting to resolve conflicting or ambiguous data.

The data set included patient demographics (age, gender, marital status, educational status), disease data (type of malignancies, metastases, and cancer treatments, co-morbidity, initial performance status assessed using the Palliative Performance Scale Adult Suandok, which was translated into Thai from the Palliative Performance Scale (PPSv2) [20]), family characteristics (number of family household members, caregivers, and caregivers’ relationship to the patients), and palliative care consultation characteristics (types of palliative consultation such as inpatient, ambulatory, and emergency department, advance care plan discussion documented by the palliative care team, and documented advance directive). We collected data on ED visits, including the number of visits, the documented reasons for the visits by the attending ED physician, the presence of symptoms evaluated by on-call palliative care physicians, and the discharge status. By reviewing the medical record and contacting the family, the principal investigator confirmed the actual location of the deceased’s death.

### Statistical analysis

The characteristics of the participants and the data on emergency department visits were presented in terms of frequency and percentage of the categorical data and mean, with standard deviation for continuous data, if the data had a normal distribution. All of the variables of interest were categorical data. Based on previous literature [21, 22], the time between the initial palliative consultation and study endpoints was categorized into three groups: 16 days, 16–100 days, and > 100 days. The logistic regression analysis was used to determine the factors associated with emergency department visits in cancer patients receiving palliative care. Variables with a *P* value of less than 0.15 from the univariate logistic regression analysis were selected to be included in the multiple logistic regression analysis [23]. Odds ratios from the multiple logistic regression were estimated with the exponential B-coefficient of each variable in order to measure the strength of association between the variables and the presence of emergency department visits among cancer patients.

All data was entered into Epidata software version 3.1 by the researcher. The STATA version 16.0 program was used to analyze the statistical data, and the level of significance was set at 0.05.

## Results

**Fig. 1:**
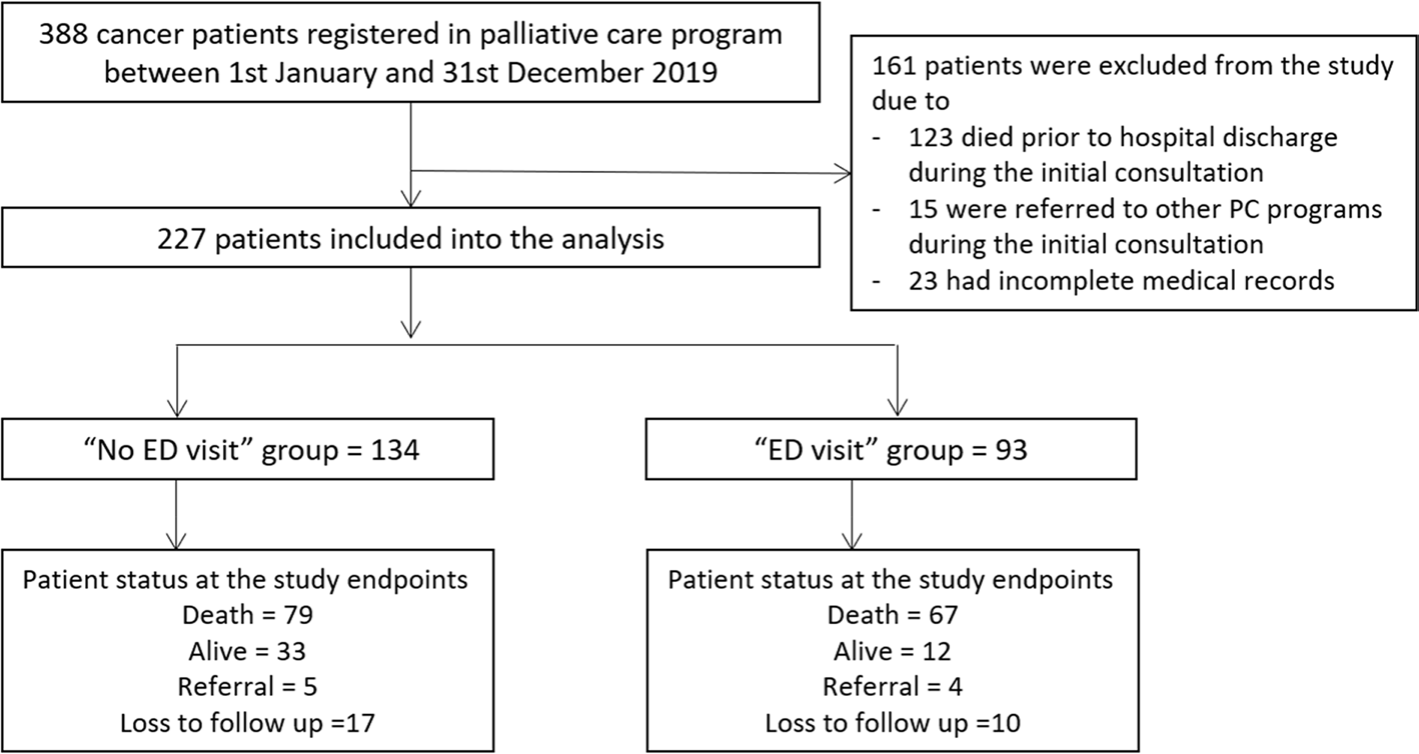
Demonstrate patient flow chart from accruing to final analysis

Three hundred and eighty-eight cancer patients enrolled in the palliative care program between January 1 and December 31, 2019. One hundred sixty-one participants were excluded from the study because 123 died prior to hospital discharge, 15 were referred to other palliative care programs, and 23 had incomplete medical records. Our study enrolled a total of 227 participants, 93 of whom visited the emergency department and the remaining 134 of whom did not. Figure 1 demonstrates the patient flow chart from accrual to final analysis.

### Demographics

The mean age was 65.5 years. The majority were men (50.7%), and the majority were married (66.1%). The majority of study participants (70.9%) had less than a bachelor’s degree. Comparing the aforementioned data, there was no statistically significant difference between patients who visited the ED and those who did not.

### Disease characteristics and comorbidities

According to the study, colorectal cancer, lung cancer, and hepatobiliary cancer were the most commonly diagnosed cancer types, accounting for 18.5, 16.3, and 11.9%, respectively. The lymph nodes (49.3%), lungs (42.2%), and liver (37.9%) were the most common sites of metastasis. The most common cancer-specific treatments received by study participants were chemotherapy (60.8%), surgery (48%), and radiotherapy (41.9%). Regarding the patient’s underlying condition, the three most common comorbidities were discovered to be hypertension (31.3%), dyslipidemia (26.9%), and diabetes (22.5%). When the disease characteristics data from the study populations were compared, no statistically significant difference between those two groups was found.

The median Palliative Performance Scale (PPS) score at the initial palliative care consultation was 50% (30–60), with 105 patients (46.3%) scoring PPS 10–40% and 122 patients (53.7%) scoring PPS 50–90%. This indicates that the proportion of patients who were bed-bound was comparable to the proportion of patients who remained independent.

### Family characteristics

In terms of housing, family, and caregivers, it was determined that the majority of patients resided in their own homes (98.2%). The majority of them (92.1%) had at least one caregiver, and the majority of their caregivers (82.8%) were family members. The majority of patients (68.3%) reside with between one and four family members. Comparing the two populations’ housing, family, and caregiver statistics revealed no statistically significant differences.

### Palliative care consultation characteristics

The majority of initial palliative care consultations occurred during inpatient, ambulatory, and emergency department care, with respective percentages of 60.8, 35.7, and 3.5%. Ninety-three percent of patients participated in discussions regarding advance care planning. Nevertheless, only 14 patients (6.2%) had advance directives seen in their medical records. Prior to the study’s endpoints, 56 patients (24.7%) received at least one home visit. Sixty-four percent of those patients received a single home visit prior to the study’s endpoints. At the end of the study, 146 patients had died, 45 were still alive, 9 had been referred to another palliative care program, and 27 were lost to follow-up. Seventy-nine study participants (34.8%) died while receiving in-patient care; sixty-one died at home, and one in the emergency room. We were unable to contact the patients’ families to identify the places of death of five study participants.

**Fig. 2:**
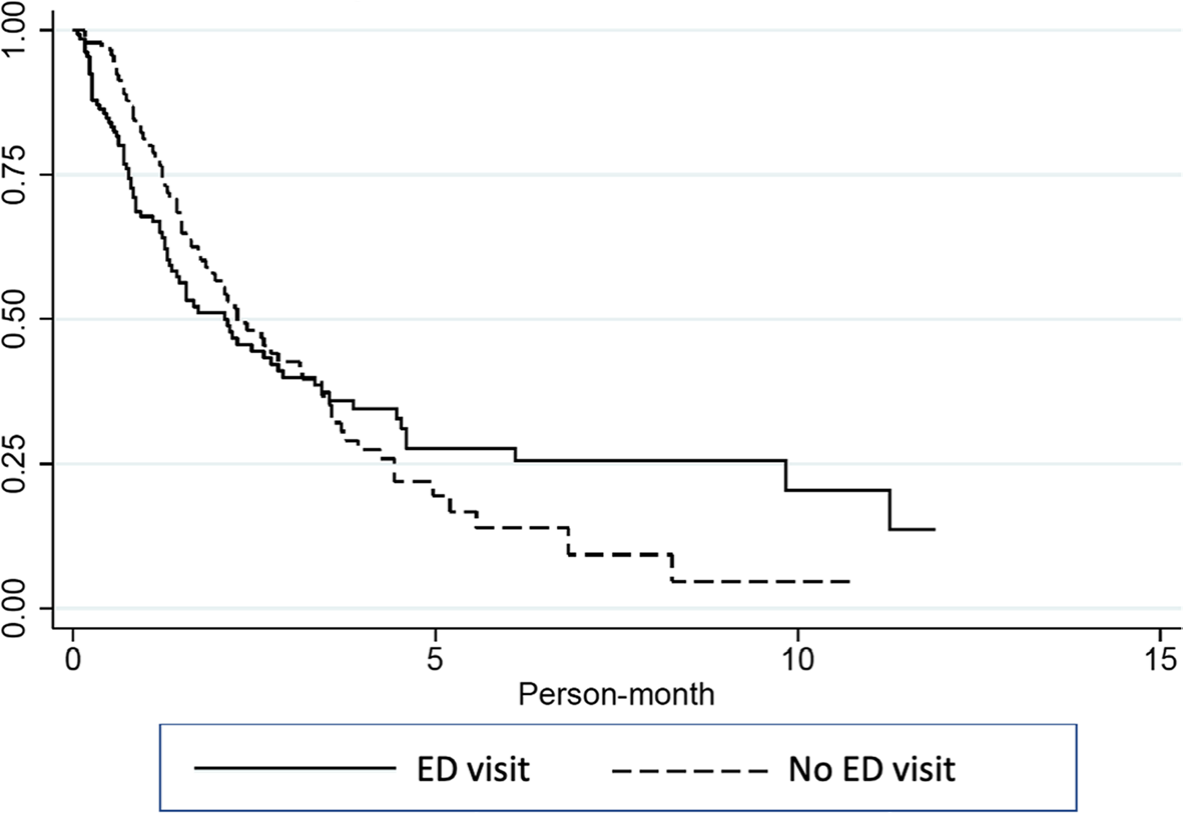
Demonstrate the Kaplan-Meier survival analysis between cancer patients who visited the ED to those who did not

The median duration between the palliative care consultation and the endpoints of the study was 36 days (IQR, 17–79), with a range of 1 to 357 days. Comparing patients who visited the ED to those who did not, the median duration of time for patients who visited the ED was 68 days (IQR, 37–134) and the median length of time for patients who did not visit the ED was 63 days (IQR, 23–182). Figure 2 depicts the results of a survival study comparing these two groups, which found no statistical significance (*p*-value = 0.88). The baseline characteristics of patients who visited the emergency department versus those who did not visit the emergency department are depicted in Table 1.

**Table 1:** The baseline characteristics of patients who visited the emergency department versus those who did not receive care at the emergency department

Characteristics	No ED visit (***N*** = 134)	ED visit (***N*** = 93)
**Demographic data**		
Age (years), mean (SD)	64.96 (15)	66.28 (14.4)
Gender		
Male, n (%)	59 (44)	56 (60.2)
Female, n (%)	75 (56)	37 (39.8)
Marital status		
Single, n (%)	22 (16.4)	21 (22.6)
Married, n (%)	92 (68.7)	58 (62.4)
Widow, n (%)	13 (9.7)	11 (11.8)
Divorced, n (%)	7 (5.2)	3 (3.2)
Education		
Uneducated, n (%)	1 (1)	2 (2.7)
Primary school, n (%)	33 (31.1)	21 (28.4)
High school, n (%)	39 (36.8)	18 (24.3)
Bachelor degree or above, n (%)	33 (31.1)	33 (44.6)
**Comorbidities**		
Hypertension, n (%)	45 (48.4)	26 (19.4)
Dyslipidemia, n (%)	33 (24.6)	28 (30.1)
Diabetes mellitus, n (%)	26 (19.4)	25 (26.9)
Cardiovascular disease, n (%)	7 (5.2)	7 (7.5)
Cerebrovascular disease, n (%)	7 (5.2)	9 (9.7)
Cirrhosis, n (%)	6 (4.5)	6 (6.4)
Dementia, n (%)	7 (5.2)	4 (4.3)
End-stage renal disease, n (%)	1 (0.7)	4 (4.3)
Parkinson’s disease, n (%)	1 (0.7)	2 (2.1)
Others, n (%)^a^	55 (41)	34 (36.6)
**Primary site of neoplasm**		
Colorectum, n (%)	25 (18.7)	17 (18.3)
Bronchus and lung, n (%)	25 (18.7)	12 (12.9)
Liver and bile duct, n (%)	15 (11.2)	12 (12.9)
Breast, n (%)	12 (9)	7 (7.5)
Head and neck, n (%)	9 (6.7)	8 (8.6)
Others, n (%) ^b^	48 (35.8)	37 (39.8)
**Metastasis**		
Lymph node, n (%)	64 (47.8)	48 (51.6)
Lung, n (%)	57 (42.5)	39 (41.9)
Liver, n (%)	48 (35.8)	38 (40.9)
Bone, n (%)	41 (30.6)	24 (25.8)
Peritoneum, n (%)	20 (14.9)	20 (21.5)
Pleura, n (%)	19 (14.2)	16 (17.2)
Brain, n (%)	11 (8.2)	10 (10.7)
Others, n (%)	23 (17.2)	20 (21.5)
**Cancer treatments**		
Chemotherapy, n (%)	79 (59)	59 (63.4)
Surgery, n (%)	63 (47)	46 (49.5)
Radiotherapy, n (%)	61 (45.5)	34 (36.6)
Targeted therapy, n (%)	13 (9.7)	9 (9.7)
Other, n (%)	6 (4.5)	3 (3.2)
PPS at the initial palliative referral mean (SD)	44.8 (17.7)	49.8 (15)
10–40%, n (%)	72 (53.73)	33 (35.48)
50–90%, n (%)	62 (46.27)	60 (64.52)
**Caregiver and family data**		
Place of living		
Home, n (%)	131 (97.8)	92 (98.9)
Nursing home, n (%)	3 (2.2)	1 (1.1)
Having caregiver(s), n (%)	124 (92.5)	85 (91.4)
Caregiver type		
Paid caregiver, n (%)	8 (6.4)	7 (8.2)
Family caregiver, n (%)	113 (91.1)	75 (88.2)
Both, n (%)	3 (2.4)	3 (3.5)
Number of caregivers		
1 person, n (%)	99 (81.8)	64 (77.1)
> 1 person, n (%)	22 (18.2)	19 (22.9)
Family household members		
1–4 persons, n (%)	89 (67.9)	66 (71.7)
> 4 persons, n (%)	42 (32.1)	26 (28.3)
**Palliative care service data**		
Place of initial palliative care consultation		
Outpatient, n (%)	43 (32.1)	38 (40.9)
Inpatient, n (%)	87 (64.9)	51 (54.8)
ED, n (%)	4 (3)	4 (4.3)
Time from initial palliative consultation to study endpoints		
< 16 days, n (%)	26 (19.4)	23 (24.7)
16–100 days, n (%)	78 (58.2)	65 (69.9)
> 100 days, n (%)	30 (22.4)	5 (5.4)
Advance care plan discussion, n (%)	126 (94)	85 (91.4)
Presence of advance directives, n (%)	9 (6.7)	5 (5.4)
Palliative home visit, n (%)	31 (23.1)	25 (26.9)
Number of home visits		
1 time, n (%)	21 (15.7)	15 (16.1)
> 1 time, n (%)	10 (7.4)	10 (10.7)
**Patient status at the study endpoints**		
Death, n (%)	79 (59)	67 (72)
Alive, n (%)	33 (24.6)	12 (12.9)
Referral, n (%)	5 (3.7)	4 (4.3)
Loss to follow-up, n (%)	17 (12.7)	10 (10.7)

^a^Other co-morbidities include Benign prostatic hyperplasia 20 participants, COPD 16 participants, Gout 12 participants, Osteoporosis 10 participants, Chronic hepatitis B infection 9 participants, Psychiatric illnesses 9 participants, Pulmonary tuberculosis 7 participants, Osteoarthritis 6 participants, and HIV 6 participants

^b^Other primary sites of neoplasm include soft tissue sarcoma in 2 patients, small bowel cancer in 2 patients, germ cell tumor in one patient, genitourinary cancer in 5 patients, malignant melanoma in 2 patients, peritoneal cancer in one patient, skin cancer in 2 patients, and unknown primary in 70 patients

### Characteristics of ED visits

In this study, a total of 93 cancer patients visited the emergency department, representing 154 ED visits. The majority of patients (58.1%) only visited the emergency department once, ranging from one to four times. The most prevalent symptoms among emergency room patients were dyspnea, fever, and pain, accounting for 21.4, 18.2, and 10.0%, respectively. Infection (20.1%), disease progression (16.2%), and gastrointestinal problems (15.6%) were the leading causes of ED visits identified by the attending ED physicians. Among 154 ED visits, 71 palliative care consultations were performed, the majority of which were undertaken during office hours (59.7%). Forty-seven percent of ED visits result in patients being discharged home after receiving care in the emergency room. Patients who required hospitalization during those visits were categorized into three groups: 52% were admitted to a palliative care unit, 43.8% to general medical wards, and 4.1% to critical care units. The characteristics of ED visits by cancer patients are detailed in Table 2.

**Table 2:** Characteristics of emergency department visits among cancer patients (*N* = 154 visits)

Characteristics	N (%)	95% CI
**Prevalence of symptoms**		
Dyspnea	33 (21.4)	15.2–28.8%
Fever	28 (18.2)	12.4–25.2%
Pain	16 (10.4)	6.1–16.3%
Alteration of conscious	15 (9.7)	5.6–15.6%
Gastrointestinal bleeding	14 (9.1)	5.1–14.8%
Nausea or vomiting	6 (3.9)	1.4–8.3%
Malaise or fatigue	5 (3.2)	1.1–7.4%
Seizure	4 (2.6)	0.7–6.5%
Others	33 (21.4)	15.2–28.8%
**Main reasons for ED visits**		
Infection	31 (20.1)	14.1–27.3%
Disease progression	25 (16.2)	10.8–23.0%
Digestive system problems	24 (15.6)	10.2–22.3%
Respiratory system problems	12 (7.8)	4.1–13.2%
CNS manifestation	9 (5.8)	2.7–10.8%
Others	23 (14.9)	9.7–21.6%
**Patient status after ED visits**		
Home	73 (47.4)	39.3–55.6%
Hospital admission	73 (47.4)	39.3–55.6%
Referral	5 (3.2)	1.1–7.4%
Dead	3 (1.9)	0.4–5.6%

### Factors associated with ED visits in cancer patients

According to the univariate logistic regression analysis, the duration of more than 100 days from the initial palliative care consultation to the study endpoints (OR 0.23; 95%CI 0.08, 0.66; *p*-value 0.01) was the factor that decreased the probability of emergency department visits among participants. PPS levels of 50–90% (OR 2.02; 95%CI 1.18, 3.47; *p*-value 0.01), on the other hand, appeared to increase the likelihood of emergency department visits. In the multiple logistic regression analysis, those two factors remained associated with ED visits: more than 100 days from the palliative care consultation to the study endpoints (OR 0.18; 95%CI 0.06, 0.55; *p*-value 0.01) and a PPS level of 50–90% (OR 2.62; 95%CI 1.44, 4.79; *p*-value 0.01). The results of the analysis are presented in Tables 3 and 4.

**Table 3:** Univariate logistic regression analysis of factors associated with ED visits

Factor	Total	ED visit	OR^**a**^	Standard error	***P***-Value	95%CI
Age ≥ 65 years	119	49	0.98	0.26	0.94	0.57, 1.66
Female gender	112	37	0.54	0.15	0.03	0.32, 0.92
Married	150	58	0.60	0.21	0.15	0.30, 1.19
Bachelor’s degree or above	66	33	0.50	0.63	0.58	0.04, 5.78
Initial PPS level 50–90%	122	60	2.02	0.56	0.01	1.18, 3.47
***Comorbidities***						
Hypertension	71	26	0.56	0.16	0.06	0.35, 1.02
Diabetes mellitus	61	28	0.67	0.22	0.21	0.36, 1.26
Dyslipidemia	51	25	0.78	0.24	0.41	0.43, 1.41
Cardiovascular disease	14	7	0.52	0.27	0.22	0.19, 1.46
Cerebrovascular disease	16	9	0.69	0.38	0.50	0.23, 2.04
Dementia	11	4	1.25	0.80	0.73	0.36, 4.40
ESRD	5	4	0.17	0.19	0.12	0.02, 1.55
***Primary site of neoplasm***						
Colorectum	42	17	0.84	0.32	0.65	0.40, 1.78
Liver and bile duct	27	12	0.98	0.44	0.98	0.41, 2.36
Breast	19	7	0.72	0.38	0.53	0.26, 2.01
Head and neck	17	8	1.10	0.59	0.86	0.39, 3.12
***Metastases***						
Lung	76	39	1.02	0.28	0.95	0.60, 1.74
Pleura	25	16	1.23	0.46	0.57	0.60, 2.54
Bone	65	24	0.84	0.25	0.57	0.47, 1.52
Lymph node	112	48	1.21	0.33	0.48	0.71, 2.05
Liver	86	38	1.20	0.33	0.51	0.70, 2.07
Peritoneum	40	20	1.53	0.53	0.23	0.77, 3.03
Brain	21	10	1.32	0.61	0.55	0.54, 3.25
***Cancer treatments***						
Chemotherapy	138	59	0.80	0.22	0.43	0.47, 1.38
Surgery	109	46	0.87	0.24	0.62	0.51, 1.48
Radiotherapy	95	34	1.39	0.38	0.24	0.81, 2.38
Targeted therapy	22	9	1.02	0.47	0.96	0.42, 2.50
***Caregiver and family data***						
Number of caregivers > 1 person	41	19	1.34	0.47	0.41	0.67, 2.66
Family household members > 4 persons	68	26	0.81	0.24	0.49	0.45, 1.46
***Palliative care service data***						
Number of palliative home visits > 1 time	36	15	0.84	0.26	0.57	0.46, 1.54
Presence of advance directives	35	5	1.29	0.74	0.66	0.42, 3.99
***Time from initial palliative consultation to study endpoints***						
16–100 days	49	23	0.94	0.31	0.86	0.49, 1.81
> 100 days	143	65	0.23	0.12	0.01	0.08, 0.66

^a^*OR* Odds ratio

**Table 4:** Multiple logistic regression analysis of factors associated ED visits

Factor	OR^**a**^	Standard error	***P***-Value	95%CI
Duration from initial palliative consultation to study endpoints > 100 days	0.18	0.10	0.01	0.06, 0.55
Female gender	0.60	0.18	0.08	0.34, 1.07
Hypertension	0.52	0.16	0.03	0.29, 0.93
ESRD	0.17	0.19	0.12	0.12, 1.55
PPS level between 50 and 90%	2.62	0.81	0.01	1.44, 4.79

^a^*OR* Odds ratio

## Discussion

Our study found that participants with > 100 days of palliative care utilized the emergency department less frequently (OR 0.23; 95%CI 0.08, 0.66; *p*-value 0.01), whereas a PPS level of 50–90% was associated with increased ED visits in cancer patients (OR 2.02; 95%CI 1.18, 3.47; *p*-value 0.01). In addition, this study found a trend that female gender was associated with fewer visits to the emergency department (OR 0.54; 95%CI 0.32, 0.92; *p*-value 0.03), although the magnitude of the association did not reach statistical significance in the multiple logistic regression analysis. This result was consistent with findings from previous retrospective cohort studies [9, 19], which indicated that older patients and men were more likely to utilize ED services at the end of life.

The results of our study showed that patient age, marital status, educational level, presence of a caregiver, and cohabitant family members were not associated with ED visits, confirming the findings of a previous cross-sectional study conducted in Italy [23]. Compared to previous research on the clinical factors associated with ED visits in the last month of life, we found no correlation between ED visits and the types of malignancies, metastases, or cancer treatments [9, 19, 24].

### Palliative care consultation characteristics

According to our study, most patients (60.8%) were referred to palliative care during their hospitalization, with a median Palliative Performance Scale of 50% during their initial palliative care consultation. In addition, patients with higher PPS levels (50–90%) were more likely to visit the emergency department. Patients with a higher PPS may have more ED visits because they are more likely to be ambulatory, allowing for ED visits, and because they are more likely to experience significant changes in their functional status, prompting a reevaluation in the ED. On the other hand, patients with a lower PPS are frequently associated with a poorer prognosis and a greater willingness to accept home care for symptom control than hospitalization. As the palliative care team was aware of the poorer prognosis for groups with lower PPS levels, they may be more proactive in providing anticipatory guidance regarding potential end-of-life symptoms than for groups with a better prognosis. This may enable the caregiver and family with the ability to manage symptoms at home if they occur.

Although some evidence suggested that advance care planning could help patients and their caregivers prepare for the end-of-life trajectory and avoid unnecessary ED visits by supporting coping with deteriorating health [25]. The majority of patients in our study (94% in the “ED visit” group versus 91.4% in the “No ED visit” group) had advance care plan discussions; therefore, we were unable to determine the effect of having advance care plans on the ED visit. In addition, a small proportion of our study participants in both groups had written advance directives, which is consistent with the results of another study conducted in a large tertiary hospital in Thailand, which revealed that only 4.9% of palliative care patients had some form of advance directive [26]. This may be due to the fact that the majority of Thai patients and their families would rather discuss their advance care plans than sign an advance directive, and would prefer for their family members to make decisions on their behalf if their condition deteriorates. This may diminish the effect of advance directives on ED visits in our study. Additionally, we found no correlation between the number of palliative home visits and ED utilization.

### Characteristics of ED visits

Prior studies have shown that the most prevalent ED diagnoses among palliative care patients are symptom-related, including abdominal pain, breathing abnormalities, nausea and vomiting, and throat and chest pain [12, 15]. Consistent with the findings of several previous studies [14, 15, 21, 27], our study revealed that uncontrolled symptoms such as dyspnea, fever, and pain, as well as disease progression and gastrointestinal issues, were among the most frequently reported reasons for emergency room visits among cancer patients. Additionally, the attending ED physician identified infection as the leading primary reason for ED visits. This is in accordance with previous studies [12] that demonstrated a high proportion of cancer patients had fever immediately prior to presentation or in the ED, resulting in hospital admission and higher antibiotic treatment rates.

### Association between palliative care consultation and ED visit

Although the evidence that palliative care interventions delivered in the hospital, at home, or in the community are more effective than usual care at reducing ED visits is not strong, based on the systematic review of the literature [22]. Nevertheless, this study suggests that longer exposure to palliative care may be associated with a decreased likelihood of emergency room visits. This association was also observed in two previous studies conducted by Rozman et al. [20] and McNamara et al. [28], which found that patients who received palliative care 3 months or more before death had a significantly lower rate of emergency department visits. Additionally, Bevins et al. also demonstrated that early palliative care referrals, defined as patients receiving palliative care within 30 days of diagnosis, were associated with fewer ED visits among patients with advanced pancreatic cancer [18].

### Limitations

This study has several limitations that should be noted. First, this was a retrospective study in which patient data was extracted from electronic databases and medical records were reviewed. Consequently, it is possible that certain data, such as the specific details of palliative care interventions, socioeconomic information, and the distance from the patient’s home to the hospital, are not completely retrieved. In our study, chart reviewers were not blinded to the outcome of interest, which may have increased the risk of bias. In addition, we did not have a second researcher enter the data twice in order to assess the inter-rater reliability of the data, which may have been susceptible to error. In addition, despite our efforts to recruit all participants enrolled in the palliative care program during the study period, the small sample size may have prevented us from identifying confounding variables that could have impacted our findings. Additionally, this study was conducted in a university hospital with more complicated patients. In other settings, such as a community hospital, results may vary. Although there were some patents that were lost to follow up in both groups, these numbers were found to be small and proportional. Consequently, this may not affect the validity of the study’s results.

## Conclusion

Our study found that advanced cancer patients who received palliative care for more than 100 days visited the emergency department less frequently. In this study, patients having a lower PPS were associated with a lower risk of ED visits. Future research may be required to fully understand how palliative care interventions reduced ED visits and to explore factors that contribute with ED visits among cancer patients with a higher PPS.

## Data Availability

All data sets on which the conclusions of the paper are based are available upon request to the corresponding author.
